# Retrosternal mass: An interesting allergic reaction to intravenous thrombolytic therapy for acute ischemic stroke

**Published:** 2013

**Authors:** Masoud Mehrpour, Mohammad Reza Motamed, Mahboubeh Aghaei, Zahra Badi

**Affiliations:** 1 Assistant Professor, Department of Neurology AND Firoozgar Hospital, Tehran University of Medical Sciences, Tehran, Iran; 2 Resident, Department of Neurology AND Firoozgar Hospital, Tehran University of Medical Sciences, Tehran, Iran

**Keywords:** Acute Ischemic Stroke, Thrombolytic Therapy, Allergic Reaction

## Abstract

Stroke is an important cause of disability and death worldwide, with the majority of strokes occurring in older people. Thrombolysis with recombinant tissue plasminogen activator (r-TPA) is the approved treatment for acute ischemic stroke. A major concern of physicians, who treat acute ischemic stroke with recombinant tissue plasminogen activator (r-TPA,) is the risk of intracerebral hemorrhage. However, other adverse reactions, including anaphylaxis and angioedema, can also occur. Here we report an interesting soft tissue reaction to intravenous r-TPA in an 80 year-old male who was treated for acute ischemic stroke.

## Introduction

Stroke is an important cause of disability and death worldwide, with the majority of strokes occurring in older people.^1, 2^ The older population has a higher mortality rate from stroke, probably due to coincidence of comorbid factors, and is more likely to require long-term care than younger patients.^3^ Thrombolysis with recombinant tissue plasminogen activator (r-TPA) is the approved treatment for acute ischemic stroke. The National Institute of Neurological Disorders and Stroke (NINDS), in a study on r-TPA stroke, found that patients treated with r-TPA had a 13% absolute increase in favorable outcome compared to placebo.^4^ A meta-analysis of thrombolytic trials by the Cochrane collaboration supports this finding.^5^

A major concern of physicians who treat acute ischemic stroke with r-TPA is the risk of intracerebral hemorrhage. However, other adverse reactions, including anaphylaxis and angioedema, can also occur. Hypersensitivity to r-TPA is uncommon and has been estimated to occur in less than 0.02% of patients who receive it for the treatment of acute myocardial infarction.^6^

Herein, we report an interesting soft tissue reaction to intravenous r-TPA in an 80 year-old male who was treated for acute ischemic stroke.

## Case Report

An 80 year-old male patient, with previous history of hypertension, was referred to Firoozgar Hospital's emergency ward 1 hour after acute onset of left hemiparesis.

At the time of stroke, he was awake, and showed no evidence of seizure. He had no previous history of head trauma, recent MI, ischemic or hemorrhagic CVA, brain surgery, or brain tumor. His blood pressure at admission was 130/85 mmHg. He was afebrile and had no respiratory distress or cardiac arrhythmia. He was awake, aware, and oriented to time, place, and person, but dysarthric. In cranial nerve examination, he had right gaze preference and flattening of the left nasolabial fold. Motor examination revealed Left-sided paresis (muscle force: 3/5). His left plantar reflex caused an upward response. Cerebellar examination of the right side was normal. His gait was not assessed.

**Figure 1:**
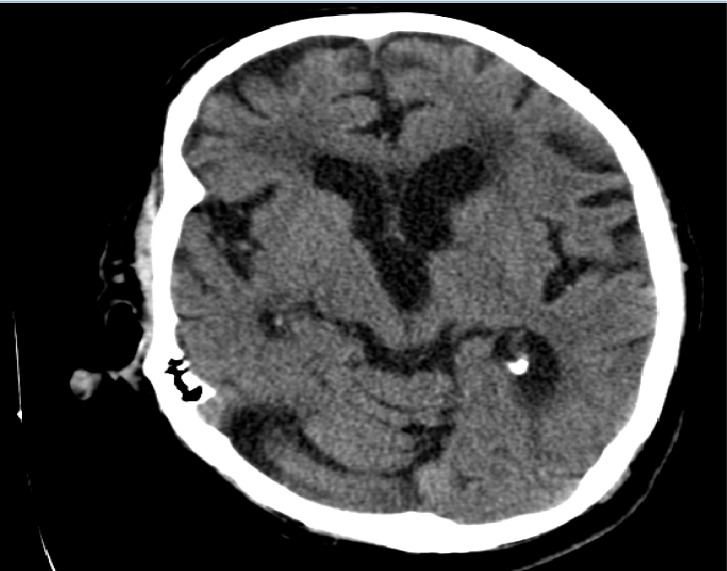
Brain CT scan of the patient at admission

With the diagnosis of cerebrovascular accident, brain CT scan without contrast was done for the patient (Figure 1).

**Figure 2:**
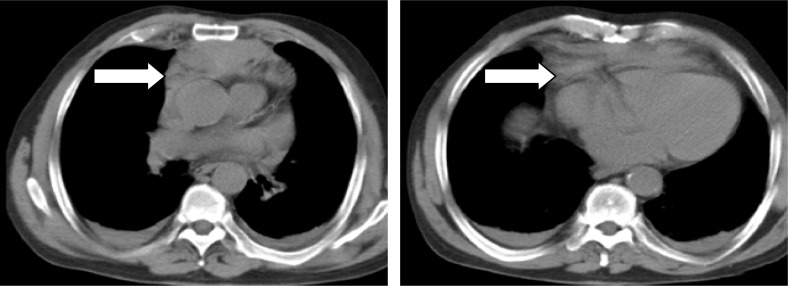
Chest CT scan of the patient after the administration of thrombolytic therapy indicating the retrosternal mass

With the diagnosis of ischemic stroke and with the use of NINDS criteria for r-TPA administration, the patient was treated with 0.9 mg/kg r-TPA.^4^ Thirty minutes after intravenous infusion of thrombolytic drug, a bluish discoloration and swelling of the skin and soft tissue appeared over the sternum. With suspicion of hemorrhagic complication, allergic reaction, and aortic dissection a chest x-ray was performed in which a mediastinal widening was seen. Spiral chest CT scan with contrast was done with the diagnosis of aortic dissection. The aorta was normal, but a retrosternal mass and mild swelling of soft tissue was seen over the sternum (Figure 2).

**Figure 3:**
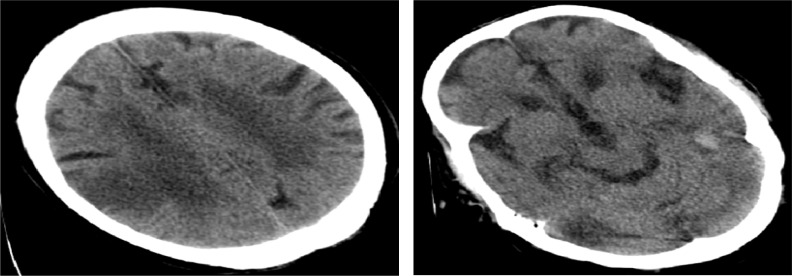
Brain CT scan after 24 hours with asymptomatic ICH

**Figure 4:**
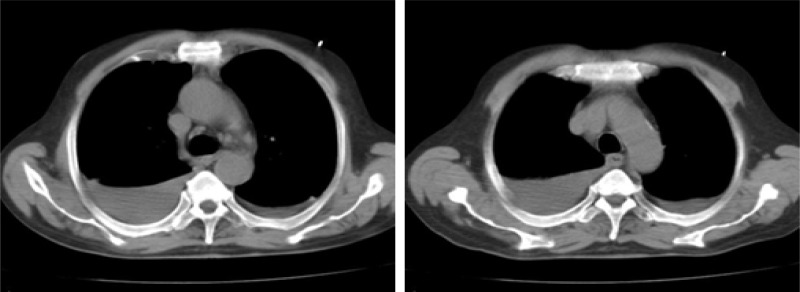
Chest CT scan after 2 weeks indicating the retrosternal mass resolved

Bluish discoloration of skin and soft tissue swelling over the sternum disappeared after 24 hours. The patient was stable until 24 hours. In the second brain CT scan, performed after 24 hours, an asymptomatic intracranial hemorrhage in the brain hemisphere contralateral to the infarcted hemisphere was detected (Figure 3). Muscle force returned to normal (5/5) around 7 days after thrombolytic therapy. Two weeks after the stroke, we consulted with a thoracic surgeon for thoracoscopy and biopsy of the retrosternal mass. Interestingly, in the second chest CT scan, before thoracoscopy, the retrosternal mass had disappeared (Figure 4). The patient was discharged with normal neurological examination.

## Discussion

Allergic reactions to thrombolytic agents can occur, but are most commonly seen with administration of streptokinase.^6^

The mechanism of allergic reaction is as follows. Plasminogen activator is a serine protease that breaks down plasminogen to plasmin. Plasmin breaks down thrombus-bound fibrin, which results in the fibrinolytic effect, and can also activate the complement cascade and kinin pathway. Bradykinin is a potent vasodilator with a short half-life due to the action of plasma kininases. The activation of complement C3 results in an amplification of the complement cascade via the alternate pathway. C3a, the cleavage product of C3, is a potent anaphylatoxin that activates mast cell degranulation and histamine release. C4a and C5a also activate mast cell degranulation. Experimental evidence suggests that anaphylaxis results in an upregulation of endogenous r-TPA levels. C3a, C4a, and C5a show a marked increase after alteplase administration. Activation of angioedema in this fashion may not necessarily lead to complement consumption and low C3 and C4 levels. Plasmin may also directly activate C1 in the classic pathway with the release of C2-kinin, another potent vasodilator thought to be responsible for angioedema in both hereditary and acquired C1 esterase inhibitor deficiency.^7, 8^

Angioedema has been reported in association with alteplase treatment of myocardial ischemia and deep vein thrombosis, but very rarely in association with alteplase treatment of acute ischemic stroke.^9^

Hill et al. reported 2 cases with allergic reaction to alteplase.^10^ One patient had severe tongue, oropharyngeal and periorbital edema and the other one had only mild swelling of tongue and lips 30-45 minutes after intravenous administration of alteplase.

Papamitsakis et al. reported another case with edema of lower lip after administration of r-TPA.^11^

In the current study, the patient had an interesting allergic reaction to r-TPA. Swelling and discoloration of soft tissue over the sternum, and skin and retrosternal swelling had not been reported in previous studies. The incidence of angioedema and other allergic reactions, including laryngeal edema, rash, and urticaria, in stroke patients treated with t-PA remains to be established. Physicians treating acute ischemic stroke with thrombolytic drugs should be aware of this infrequent complication. In conclusion, we suggest the inspection of patients’ skin, tongue, and oropharynx 30-45 minutes after the start of a thrombolytic infusion for possible uncommon reactions.
